# Gamma-glutamyl metformin inhibits renal cell carcinoma progression by activating AMPK signaling pathway

**DOI:** 10.3389/fphar.2026.1807987

**Published:** 2026-05-07

**Authors:** Zheng Lv, Li Song Zhang, Awuti Aisha, Tian Hang Wang, Hai Yan Cui, Shuai Tang, Jiang Hui Zhang, Fan Chang, Wen Song Wu, Lu Yuan Li, Zhi Song Zhang, Fang Min Chen

**Affiliations:** 1 Department of Urology, Central Hospital, Tianjin University/Tianjin Third Central Hospital, Tianjin, China; 2 Tianjin Key Laboratory of Extracorporeal Life Support for Critical Diseases, Tianjin, China; 3 State Key Laboratory of Medicinal Chemical Biology, College of Pharmacy and Tianjin Key Laboratory of Molecular Drug Research, Nankai University, Tianjin, China; 4 The Third Central Clinical College of Tianjin Medical University, Tianjin, China; 5 Department of Urology, Affiliated Hospital of Jianghan University, Wuhan, China

**Keywords:** AMPK signaling pathway, gamma-glutamyl transpeptidase, metformin, prodrug, renal cancer

## Abstract

Metformin is a promising candidate for the treatment of renal cell carcinoma (RCC), and elucidating its anti-tumor mechanisms is of great clinical significance. Overexpression of Gamma-glutamyl transpeptidase (GGT) in RCC tissues has been identified as a potential biomarker for renal cell tumors. Thus, there is an urgent need to develop a metformin prodrug that leverages the overexpression specificity of GGT in RCC tissues to achieve targeted release of metformin for RCC therapy. Herein, we innovatively report the development and the antitumor activity of gamma-Glutamyl Metformin (γE-Met), which exerts its effects by activating AMP-activated protein kinase (AMPK) signaling pathway. Compared with metformin, γE-Met specifically releases free metformin in renal cancer cells under the catalysis of GGT, thereby prolonging the retention time of metformin in tumor cells and more effectively inhibiting the growth and metastasis of renal cancer cells. Our experimental results demonstrate that GGT-responsive γE-Met can significantly enhance the therapeutic efficacy of metformin, rendering it a promising metformin prodrug with substantial clinical application prospects.

## Introduction

Renal cell carcinoma (RCC) represents the most prevalent malignant neoplasm of the kidney. Despite advances in targeted and immunotherapeutic strategies, a substantial proportion of patients with advanced or metastatic RCC develop resistance to treatment. The inherent heterogeneity of RCC highlights the urgent need for novel therapeutic approaches. Adenosine monophosphate-activated protein kinase (AMPK) acts as a central cellular energy sensor and master regulator of metabolism. Its activation shifts cellular processes from anabolism to catabolism, thereby restoring intracellular energy homeostasis. In oncology, AMPK activation is known to exert potent tumor-suppressive effects, including the inhibition of key drivers of cell proliferation. Pharmacological activation of AMPK has thus emerged as a promising therapeutic strategy for various cancers, including RCC, where aberrant metabolic reprogramming is a defining hallmark (Peng et al., 2023).

Metformin (Met), a biguanide-class drug, has been used for the treatment of type 2 diabetes mellitus for decades (Yu and Suissa, 2023). Mechanistically, Met inhibits mitochondrial complex I, which in turn triggers AMPK activation (Li et al., 2019). Previous studies have demonstrated that Met exerts anti-tumor effects by inhibiting mitochondrial respiratory chain complex I and activating the AMPK signaling pathways in tumor cells (Wang et al., 2019), and it has been shown to reduce tumor growth by 25% in mice (Chun and Kim, 2021; Zhou et al., 2023). However, the clinical translation of Met as an anti-cancer agent has been hampered by its relatively low potency, as therapeutic anti-tumor efficacy typically requires high concentrations that are often clinically unattainable. Preclinical studies have reported that the high doses of Met used in rat models (250 mg/kg) exceed the typical clinical doses administered to humans (Foretz et al., 2014). This discrepancy raises important questions about the translatability of animal study findings to human RCC treatment.

To overcome these limitations, prodrug strategies have been explored to enhance the delivery, bioavailability, and tumor-targeting capacity of therapeutic agents. Gamma-glutamylation is one such strategy, involving the conjugation of a gamma-glutamyl moiety to an active drug molecule (Mitri and Castellano, 2023). This modification can exploit the elevated activity of gamma-glutamyl transpeptidase (GGT), an enzyme frequently overexpressed on the surface of various cancer cells, including RCC, to enable tumor-selective activation of the prodrug. Additionally, it has been demonstrated that the upregulation of GGT in cancer cells triggers glutathione-mediated γ-glutamyl transfer reactions, facilitating efficient tumor penetration of glutathione-modified nanocarriers (Yu et al., 2020) or nanocarriers containing γ-glutamyl moieties (Wang et al., 2020). The gamma-glutamyl derivative of Met (gamma-glutamyl Met, γE-Met) is a rational chemical innovation designed to improve the pharmacokinetic properties and tumor-specific delivery of Met.

In this report, we hypothesize that γE-Met may exhibit superior anti-tumor efficacy against RCC compared to the parent compound Met, potentially *via* enhanced tumor-specific accumulation and more robust activation of the AMPK signaling pathway. Our findings aim to provide a preclinical foundation for evaluating γE-Met as a novel metabolism-targeted prodrug candidate for the treatment of RCC.

## Materials and methods

### Materials

Tumor samples were collected from 125 RCC patients who underwent nephrectomy at the Urology Department of Central hospital, Tianjin University (Tianjin, China) from January 2018 to January 2024. Clinicopathologic characteristics of the patients are summarized in Supplementary Table 1. All patients provided written informed consent for study participation, and the study protocol was approved by the ethics committee of Central hospital, Tianjin University (IRB2023-010-01).

### Cell culture

293T were cultured in high-glucose Dulbecco’s Modified Eagle Medium (DMEM) supplemented with fetal bovine serum (FBS) and 1% penicillin-streptomycin (PS). HK-2, Caki-1, 769P and 786O were cultured in RPMI 1640 medium containing 10% FBS and 1% PS. Renca cells were cultured in RPMI 1640 medium supplemented with 1% sodium pyruvate, 1% non-essential amino acid, 10% FBS and 1% PS. ACHN were cultured in Minimum Essential Medium (MEM) with 10% FBS and 1% PS. All cell lines were incubated in a humidified incubator with 5% CO_2_ at 37 °C.

### *In Vivo* near infrared fluorescence imaging of GGT

To assess the *in vivo* distribution of GGT protein in a murine RCC model, the fluorescent probe γE-DMN (100 μM) in 150 μL phosphate-buffered saline (PBS) was intravenously injected into Renca tumor-bearing Balb/C mice. The specificity of γE-DMN for *in vivo* detection of GGT in tumor tissue has been previously validated in murine model (Liu et al., 2023). Real-time whole-body fluorescence imaging was performed using an IVIS fluorescence imaging system. For GGT inhibition controls, GGsTop (5 mM, 100 μL), a specific GGT inhibitor, was intratumorally injected prior to intravenous γE-DMN administration to suppress GGT activity in Renca tumors. Mice were euthanized 4–6 h post-injection, the Renca tumors and major organs (heart, lungs, liver, spleen, and kidneys) were harvested for IVIS fluorescence imaging.

### *In Vitro* γE-Met release

The release of Met from γE-Met under GGT catalysis was first assessed in cell-free condition. γE-Met was incubated with different concentrations of GGT at 37° C for 120 min, and Met release was quantified. At a γE-Met concentration of 25 μM, incubation with 20 mU/mL GGT resulted in maximal Met release, indicating that GGT can effectively mediate the complete release of Met from γE-Met in this cell-free system.

### Intracellular drug release

293T cells were seeded onto glass bottom dish (1 × 10^6^ cells per dish) and cultured overnight. GGT plasmid was transfected into 293T cells. After 48 h, γE-Met was added to the culture medium for 0.5–4 h. After freeze-drying the supernatant, protein-insoluble substances were dissolved it in acetone. Acetone was removed by rotary evaporation, and the sample was dissolved in acetonitrile for HPLC detection. The HPLC results showed that in 293T cells with high expression of GGT, γE-Met can release free Met.

### Cell viability analysis

*In vitro* cell proliferation was evaluated using a CCK8 assay. Cells were seeded into 96-well plates at a density of 1.0 × 10^4^ cells per well in 200 μL culture medium and incubated at 37 °C for 16 h. The culture medium was then replaced with fresh medium containing various concentrations of Met, γE-Met, and cells were co-incubated for 24, 48 and 72 h. Untreated cells served as the control group. Absorbance at 490 nm was measured using a VICTOR NIVO 5F Multimode plate reader (Revvity, USA). For *in vitro* GGT inhibition studies, 786O and ACHN cells were pretreated with 50 μM GGsTop for 2 h prior to the addition of γE-Met or Met, and GGsTop (50 μM) was maintained in the medium for the duration of the assay. For *in vitro* AMPK inhibition studies, cells were pretreated with or without 10 μM Compound C (a specific AMPK inhibitor) for 1 h, followed by treatment with 2 mM γE-Met for 48 h.

### Scratch and invasion assays

Scratch and invasion assays were performed using previously reported protocols. For scratch assays, the distance between scratch edges was photographed and measured using an Olympus DX31 microscope (×200 magnification) at 0 and 12 h post-scratching. For invasion assays, 5 × 10^4^ cells were seeded into the Matrigel-coated upper chamber of a Transwell insert (3422, Costar; Matrigel, 356,224, BD Biosciences). For GGT inhibition studies, fresh medium containing 2 mM γE-Met with or without 50 μM GGsTop was added to the lower chamber. For AMPK inhibition studies, cells were pretreated with or without 10 μM Compound C for 1 h prior to seeding and treatment with 2 mM γE-Met. After 12 h of incubation, all cells were fixed with 4% paraformaldehyde, and the migrated cells were stained with 0.5% crystal violet (C8470, Solarbio, China).

### Western blot (WB) assays

Western blot analysis was performed according to previously established protocols. The information of antibodies is listed in Supplementary Table 2. Relative protein expression levels were quantified using ImageJ software and calculated as the ratio of the gray value of the target protein band to the internal control β-actin band.

### Hematoxylin-eosin (HE) staining and immunochemistry (IHC) assays

4-μm paraffin sections of fixed tissues were stained with HE to evaluate morphological changes in tumors, metastatic lung foci, and normal tissues (liver, spleen, heart, contralateral kidney). For IHC assays, sections were incubated with a GGT-specific antibody at 4 °C overnight and detected using the IHC-SP method. Stain intensity was scored as negative (0), weak (1), moderate (2), and strong (3). Staining intensity was scored as 1 (0%–10%), 2 (11%–20%), 3 (21%–50%) and 4 (51%–100%). The total IHC score was calculated by multiplying the intensity and extent scores (range: 0-12), with a score <2 defined as negative staining and 2–12 defined as positive staining.

### *In Vivo* antitumor activity evaluation

6-8-week-old BALB/c female mice were used to establish an orthotopic allograft RCC model. Renca cells (2 × 10^5^) suspended in 50 μL sterilized PBS/Matrigel (1:1) were injected into the subcapsular space of the right kidney under isoflurane inhalation anesthesia (4% induction, 1.5%–2% maintenance). All animal procedures were approved by the Animal Ethics Committee of Central hospital, Tianjin University. The sample size for *in vivo* efficacy studies was determined based on preliminary experiments and power analysis using G*Power software (version 3.1, Heinrich-Heine-Universität Düsseldorf, Germany). One week after tumor implantation, 50 mice were randomly divided into five group (10 mice per group) and treated by intraperitoneal injection every 2 days for 4 weeks as follows: normal saline (NC group), Met 50 mg/kg, γE-Met 50 mg/kg, Met 100 mg/kg, and γE-Met 100 mg/kg. To validate the GGT-dependency of γE-Met’s antitumor efficacy, an additional cohort of orthotopic Renca tumor-bearing mice was established and randomly divided into two groups 1-week post-implantation: (1) γE-Met monotherapy (50 mg/kg); (2) γE-Met (50 mg/kg) +GGsTop (10 mg/kg). GGsTop was administered intraperitoneally 2 h prior to each γE-Met injection, and treatments were continued every 2 days for 4 weeks. At the end of the experiment, all mice were euthanized under 3% sodium pentobarbital anesthesia. Primary tumors, contralateral kidneys, and major organs (heart, spleen, liver, lung) were harvested, weighed, measured, photographed, and fixed for subsequent HE and IHC analysis. All animals were housed and managed in accordance with the ethical standards established by the Tianjin Committee on the Use and Care of Laboratory Animals.

### Statistical analysis

All statistical analyses were performed using GraphPad Prism 9.0 software (GraphPad Software, Inc., USA). Quantitative data are presented as the mean ± standard deviation. Student’s t-test was used for comparisons between two groups, and one-way analysis of variance (ANOVA) followed by Tukey’s *post hoc* test was applied for multiple group comparisons. The Kaplan-Meier method with the Log-rank test was used to analyze patient survival differences stratified by GGT expression. Univariate and multivariate Cox proportional hazards regression models were used to identify independent prognostic factors for renal cell carcinoma (RCC) patients. A two-tailed *p*-value <0.05 was considered statistically significant for all analyses.

## Results

### Clinical significance of GGT in RCC and in vivo detection of GGT in murine tumor tissues

**FIGURE 1:**
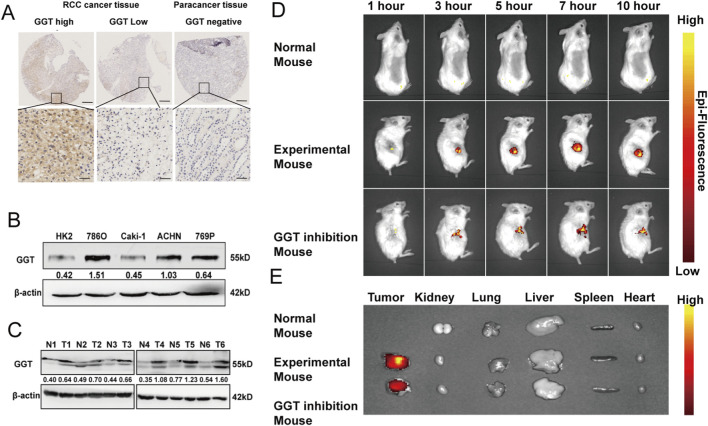
**(A)** IHC analysis of GGT expression in RCC cancer tissues and paracancer tissues. **(B)** WB analysis of GGT expression in one normal human proximal tubule epithelial cell line (HK2) and four renal cancer cell lines (786O, ACHN, Caki-1 and 769P). **(C)** WB analysis of GGT expression in six paired RCC cancer tissues and paracancer tissues. **(D)** Real-time fluorescence images of Renca tumor xenograft mice before intravenous injection of γE-DMN after intravenous injection of designated probe or GGsTop (5 mM, 100μL). 1: Normal mouse, 2: Experimental mouse, 3: GGT inhibition mouse. **(E)** Fluorescence images of tumors and major organs 4-6 h after intravenous injection of γE-DMN or γE-DMN and GGsTop. 1: Normal mouse, 2: Experimental mouse, 3: GGT inhibition mouse.

GGT protein was overexpressed in RCC cancer tissues compared with paracancer normal tissues (Figure 1A-C). Among RCC cell lines, GGT was moderately to highly expressed in 786O, ACHN and 769P cells relative to HK-2 and Caki-1 cells (Figure 1B). As shown in Supplementary Table 1, high GGT expression was associated with clinicopathological features including age >60 years, T3-T4 stage, M1 stage, and advanced AJCC stage in RCC patients. Additionally, high GGT expression was significantly correlated with poorer overall survival (OS; Log Rank = 24.349, *P* < 0.001) and progression-free survival (PFS; Log Rank = 34.795, *P* < 0.001) in RCC patients (Supplementary Figure S1B,C), and multivariate analysis identified GGT expression as an independent prognostic factor for OS and PFS in RCC patients (Supplementary Table 3).

In tumor-bearing Balb/C mice, intravenous injection of γE-DMN resulted in gradually increasing near-infrared (NIR) fluorescence at the renal tumor site, with maximal fluorescence observed at 4–6 h post-injection. Intratumoral pretreatment with the GGT inhibitor GGsTop effectively abrogated this tumor-specific NIR fluorescence, with fluorescence intensity 3–4 times lower than that in untreated mice at 4–6 h. Tumor tissues in both groups exhibited NIR fluorescence signals that increased over time and peaked at approximately 3 h post-injection, confirming the ability of γE-DMN to specifically recognize GGT in tumor tissue *in vivo*. The NIR fluorescence signal in untreated mice was approximately 5.21 times higher than that in GGsTop-treated mice (Figure 1D), and strong NIR fluorescence was detected at the renal tumor site in γE-DMN-injected mice (Figure 1E), consistent with previous observations of high GGT activity in murine RCC tumor tissue. Collectively, these results demonstrate that the GGT-recognizable NIR fluorescent probe γE-DMN can effectively distinguish GGT-positive tumor cells from normal cells both *in vitro* and *in vivo*.

### Preparations and characterizations of γE-Met

**FIGURE 2:**
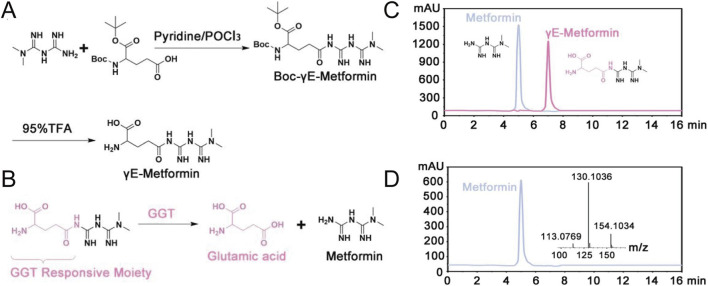
**(A)** Schematic of the preparation process of γE-Met. **(B)** The mechanism of γE-Met cleaved by GGT to release free metformin. **(C)** A High-performance liquid chromatography spectrum of γE-Met and Met in cell-free condition. **(D)** High resolution mass spectrometry of γE-Met releasing Met in 293T cell.

**FIGURE 3:**
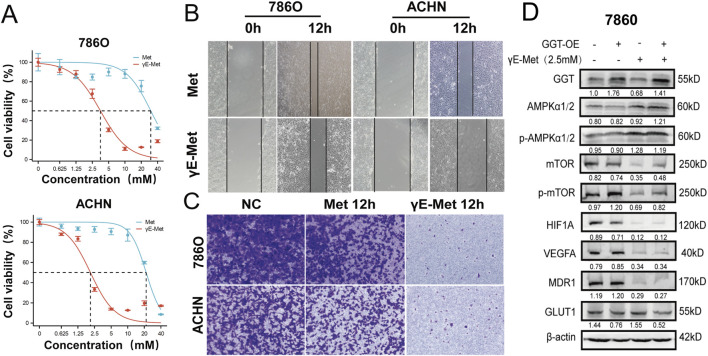
**(A)** Renal cancer cells were incubated with Met and γE-Met for 24 h and then subjected to CCK8 assays. **(B)** Representative images (200× magnification) of scratch migration assay showing 786O and ACHN cells treated with Met (10 mM) or γE-Met (2 mM) for 12 h. Scale bar=100μm. **(C)** Representative images (200× magnification) of Transwell invasion assay showing 786O and ACHN cells treated with Met (10 mM) or γE-Met (2 mM) for 12 h. Invading cells were stained with crystal violet. Scale bar = 50 μm. **(D)** Representative Western blot images showing expression of AMPK, p-AMPK (Thr172), mTOR, p-mTOR (Ser2448), HIF1A, VEGFA, MDR1, GLUT1, and β-actin in 786O and ACHN cells treated with Met (10 mM) or γE-Met (2 mM) for 24 h.

All compounds used in this study were purified by HPLC to a purity of >98%. γE-Met exhibited good chemical stability under physiological buffer conditions (Supplementary Figure S1). The synthetic route of γE-Met is detailed in Figure 2A and Supporting Information (S2), with γE-Met synthesized in two steps with an overall yield of 34.35%. Trifluoroacetic acid and dichloromethane were removed from the reaction mixture by rotary evaporation, and the crude product was purified by HPLC to obtain γE-Met as a white solid (1.36 g, 52.71% yield for the final step). The chemical structures of the intermediate Boc-γE-Met (Supplementary Figures S2-3) and the final product γE-Met were fully characterized by ^1^H NMR, ^13^C NMR and high-resolution mass spectrometry (HRMS) (Supplementary Figures S4-7). Incubation of γE-Met with increasing concentrations of GGT at 37 °C for 120 min resulted in a concentration-dependent increase in Met release (Figure 2B, C), with maximal release observed at 20 mU/mL GGT for 25 μM γE-Met. γE-Met showed negligible UV–vis absorption in the presence of biological substances, confirming its high specificity for GGT (Supplementary Figure S4). HPLC analysis of GGT-overexpressing 293T cells incubated with γE-Met revealed that γE-Met (retention time [TR] = 6.56 min) was completely converted to free Met (TR = 5.78 min) within 3 h (Figure 3D), further confirming that GGT can mediate intracellular Met release from γE-Met.

### *In Vivo* renal cancer-targeting effect of γE-Met *via* the AMPK signaling pathway

786O, ACHN were exposed to incremental concentrations of Met or γE-Met for 24h. Met inhibited cell growth in a dose-dependent manner with IC_50_ values of 29.98 mM (786O) and 21.61 mM (ACHN), while γE-Met exhibited more potent dose-dependent growth inhibition with IC_50_ values of 3.53 mM (786O) and 2.11 mM (ACHN) (Figure 3A). In addition, γE-Met showed stronger inhibitory effects on the migration (Figures 3B; Supplementary Figure S8A) and invasion (Figures 3C; Supplementary Figure S8B) of 786O and ACHN cells compared with Met. Western blot analysis revealed that γE-Met upregulated the expression of AMPK and phosphorylated AMPK (p-AMPK) and downregulated the expression of mTOR, p-mTOR, HIF1A, VEGFA, MDR1 and GLUT1 in RCC cells (Figure 3D; Supplementary Figure S9). Densitometric quantification confirmed significantly higher p-AMPK/AMPK ratios in γE-Met-treated cells (p < 0.05 vs. control) (Supplementary Figure S9). GGT overexpression in 786O and ACHN cells further enhanced the ability of γE-Met to activate the AMPK signaling pathway, and CCK-8, scratch and invasion assays confirmed that GGT overexpression enabled γE-Met to inhibit RCC cell proliferation, migration and invasion more effectively than in empty vector-transfected cells. These results suggest that γE-Met may exert anti-tumor activity in RCC cells *via* activation of the AMPK signaling pathway.

### *In Vivo* antitumor effect of γE-Met

**FIGURE 4:**
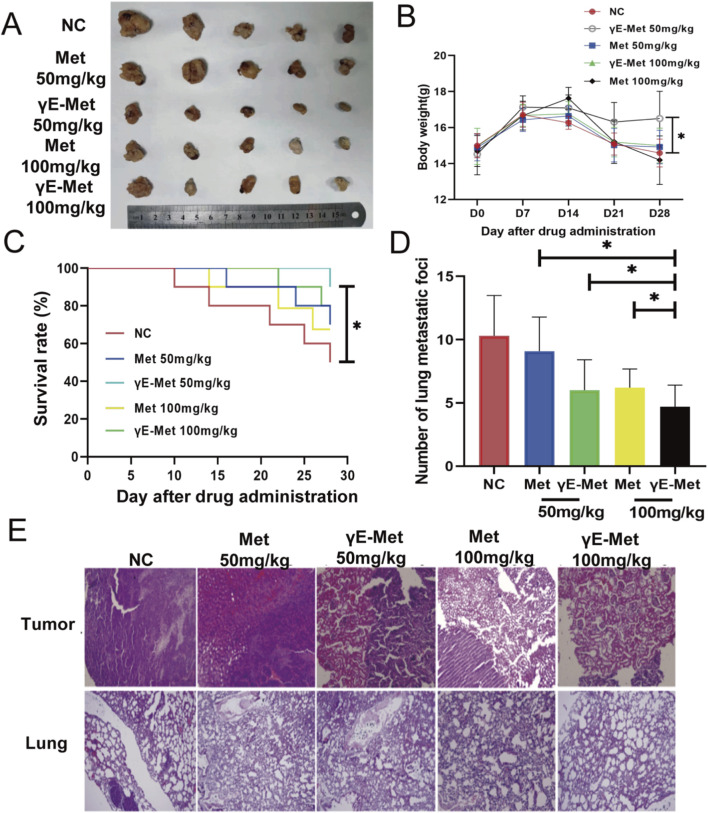
**(A)** Gross appearance of kidney tumors in groups of NC, Met 50 mg/kg, γE-Met 50 mg/kg, Met 100 mg/kg and γE-Met 100 mg/kg. **(B)** The change of body weight of mice by line chart. *p < 0.05. **(C)** The survival rates of mice in each group were compared by Kaplan-Meier analysis. *p < 0.05. **(D)** The number of lung metastatic foci were compared. *p < 0.05. **(E)** Pathological changes in the tumor and lung were detected by HE staining.

In the orthotopic murine RCC model, treatment with 100 mg/kg Met and 100 mg/kg γE-Met both significantly inhibited *in vivo* tumor growth (Figure 4A). Notably, mice in the 50 mg/kg γE-Met group had a higher body weight than those in the NC group at the end of the experiment (Figure 4B), indicating good tolerability of γE-Met at this dose. In addition, 50 mg/kg γE-Met exhibited more effective tumor growth inhibition compared with 50 mg/kg Met, and the survival rate of mice in the 100 mg/kg γE-Met group was higher than that in the NC group (Figure 4C). The number of lung metastatic foci was also lower in the 50 mg/kg γE-Met group compared with the 50 mg/kg Met group (Figures 4D; Supplementary Figure S10). HE staining revealed irregular growth of renal tumors, while no significant morphological alterations were observed in the major organs (liver, kidney, lung, heart) of treated mice (Figures 4E; Supplementary Figure S11), further confirming the *in vivo* safety of γE-Met. Met released from γE-Met maintained a longer residence time in tumor tissue at the same dose compared with direct Met administration (Supplementary Figure S12-13), and the intratumoral concentration of Met at 24 h post-γE-Met injection was approximately 3-fold higher than that post-Met injection (Supplementary Figure S14).

### GGT activity and AMPK activation are required for γe-Met-mediated antitumor activity

*In vitro* pretreatment with GGsTop significantly attenuated γE-Met-induced inhibition of proliferation, migration and invasion in 786O and ACHN cells and abrogated γE-Met-mediated AMPK phosphorylation and downstream signaling modulation (Supplementary Figure S15-S18). *In vivo*, co-administration of GGsTop with γE-Met significantly reduced antitumor efficacy of γE-Met in the orthotopic Renca model (Supplementary Figure S19). These data provide evidence that the antitumor effects of γE-Met are likely mediated *via* GGT-dependent activation. Similarly, pretreatment with Compound C (an AMPK inhibitor) significantly abrogated γE-Met-induced inhibition of RCC cell proliferation, migration and invasion (Supplementary Figure S15-S17), and Western blot analysis confirmed that Compound C blocked γE-Met-mediated AMPK phosphorylation and reversed the inhibition of mTOR signaling (Supplementary Figure S18). These results suggest that AMPK activation is a key mediator of γE-Met’s *in vitro* anti-tumor activity in RCC cells.

## Discussion

In this report, we present preclinical evidence that γE-Met, a GGT-targeted prodrug of Met, exhibits enhanced anti-proliferative effects against RCC cells *in vitro* and more potent *in vivo* tumor growth inhibition, compared with the parent compound Met. Our findings also suggest that this improved efficacy may be linked to the more robust and sustained activation of the AMPK signaling pathway by γE-Met in RCC cells.

GGT is a pivotal member of the N-terminal nucleophilic hydrolytic enzyme family and regulates multiple physiological processes, including the intracellular glutamyl cycle, cysteine metabolism and cellular redox homeostasis (Whitfield, 2001). Elevated serum GGT levels have been reported in 70% of patients with metastatic RCC, and GGT has been proposed as a potential biomarker for carcinogenesis, tumor progression and poor prognosis in RCC (Takemura et al., 2020). Our study confirmed GGT upregulation in RCC tumor tissues and cell lines, and further demonstrated that high GGT expression is associated with unfavorable clinicopathological characteristics and poorer prognosis in RCC patients. GGT overexpression in cancer cells can cleave extracellular glutathione, leading to reactive oxygen species production and activation of the pro-tumorigenic NF-kB pathway (Mitri and Castellano, 2023), which may contribute to the aggressive clinical behavior of GGT-high RCC.

Based on the tumor-specific overexpression of GGT in RCC, we developed γE-Met as a novel GGT-targeted prodrug for RCC treatment. Our data suggest that after accumulating in RCC tissues, γE-Met is recognized and cleaved by GGT to release free Met, and γE-Met treatment results in significantly higher intracellular Met levels in RCC cells compared with direct Met administration at the same dose. Additionally, γE-Met exhibits a prolonged residence time in RCC cells, which may be attributed to the gamma-glutamyl modification altering the cellular accumulation and secretion of the drug. Our *in vivo* findings are consistent with our *in vitro* results, with γE-Met showing superior anti-tumor efficacy compared with Met in the orthotopic murine RCC model. This improved potency is likely due to the prodrug design of γE-Met: the conjugation of a gamma-glutamyl moiety may alter the pharmacokinetic profile of Met by enhancing cellular uptake or reducing renal clearance (Li et al., 2024). More importantly, GGT overexpression on the surface of RCC cells provides a tumor-selective activation mechanism for γE-Met, which we hypothesize mediates the intracellular release of active Met at the tumor site, leading to higher local drug concentrations and reduced systemic exposure. This targeted delivery strategy addresses a major limitation of conventional Met therapy for cancer—the need for high, potentially toxic doses to achieve effective intratumoral drug levels (Wen et al., 2022; Jafarzadeh et al., 2022).

To validate the GGT-dependency of γE-Met’s efficacy, we performed *in vitro* and *in vivo* GGT inhibition experiments using GGsTop. Our results showed that GGsTop pretreatment significantly attenuated γE-Met’s anti-tumor effects and abrogated its ability to activate the AMPK signaling pathway, providing direct evidence that γE-Met’s efficacy is likely mediated *via* GGT-dependent cleavage and Met release. This GGT dependency not only validates our proposed tumor-selective activation mechanism but also suggests that γE-Met may be more effective in patients with GGT-high RCC, supporting the potential of GGT as a predictive biomarker for patient stratification in future clinical studies. We also used Compound C to investigate the role of AMPK activation in γE-Met’s anti-tumor effects, and found that AMPK inhibition blocked γE-Met-induced suppression of RCC cell proliferation and migration, as well as γE-Met-mediated modulation of the mTOR signaling pathway. These findings suggest that AMPK activation is a key functional mediator of γE-Met’s anti-tumor activity in RCC cells.

Previous studies have demonstrated that GGT overexpression in cancer cells can facilitate the deep tumor penetration of nanocarriers containing γ-glutamyl moieties (Fu et al., 2023). Our results extend this finding to small-molecule prodrugs by showing that γE-Met, a γ-glutamyl-modified small molecule, exhibits tumor-selective accumulation and enhanced anti-tumor efficacy in RCC. At the molecular level, our data suggest that AMPK activation is a central mediator of γE-Met 's effects in RCC: γE-Met induced a more pronounced phosphorylation of AMPK compared with Met, and this amplified AMPK signal led to potent inhibition of the mTORC1 pathway, a key driver of cell growth and proliferation that is frequently dysregulated in RCC (Kang et al., 2024; Dodd et al., 2015). The resulting inhibition of RCC cell proliferation, migration and invasion is consistent with the known tumor-suppressive effects of AMPK activation, further supporting the role of the AMPK-mTOR pathway in mediating γE-Met’s anti-tumor activity.

This study has several limitations that should be acknowledged. First, our investigations were primarily conducted using established RCC cell lines. Future studies using patient-derived organoids or primary RCC cells would help to strengthen the clinical translatability of our findings. Second, our mechanistic analysis focused on the AMPK-mTOR axis, and we did not evaluate other key AMPK substrates such as acetyl-CoA carboxylase (ACC), a critical mediator of AMPK-regulated lipid metabolism. Assessing the phosphorylation of ACC and other downstream AMPK targets would provide a more comprehensive understanding of the metabolic effects of γE-Met in RCC and is an important direction for future research. Third, the *in vitro* IC_50_ values of γE-Met for RCC cells remain in the millimolar range (approximately 2–4 mM), which is a key consideration for clinical translation. However, γE-Met exhibited an approximately 8–10-fold improvement in in vitro potency compared with Met, and the robust *in vivo* efficacy observed at well-tolerated doses (50–100 mg/kg) suggests that the millimolar *in vitro* IC_50_ values may overestimate the concentrations required for therapeutic efficacy *in vivo*, where stromal and immune cell interactions, as well as tumor-specific pharmacokinetics, may enhance drug activity.

Our preclinical findings highlight the clinical significance of GGT overexpression in RCC and its association with poor prognosis, and we successfully developed γE-Met, a GGT-recognizable Met prodrug that is selectively cleaved to release free Met in GGT-positive RCC cells. Our results suggest that γE-Met may exert more potent anti-tumor effects against RCC compared with Met, potentially *via* enhanced tumor-specific accumulation and robust activation of the AMPK signaling pathway. Collectively, these preclinical data provide a rationale for the further development of γE-Met as a targeted metabolic therapy for RCC, and potentially for other GGT-positive, metabolism-dependent cancers.

## Data Availability

The raw data supporting the conclusions of this article will be made available by the authors, without undue reservation.

## References

[B1] Bahadoram S. Davoodi M. Hassanzadeh S. Bahadoram M. Barahman M. Mafakher L. (2022). Renal cell carcinoma: an overview of the epidemiology, diagnosis, and treatment. G. Ital. Nefrol. organo Uff. della Soc. Ital. Nefrol. 39 (3), 2022-vol3. 35819037

[B2] Chun Y. Kim J. (2021). AMPK-mTOR signaling and cellular adaptations in hypoxia. Int. Journal Molecular Sciences 22 (18), 9765. 10.3390/ijms22189765 34575924 PMC8465282

[B3] Dodd K. M. Yang J. Shen M. H. Sampson J. R. Tee A. R. (2015). mTORC1 drives HIF-1α and VEGF-A signalling *via* multiple mechanisms involving 4E-BP1, S6K1 and STAT3. Oncogene 34 (17), 2239–2250. 10.1038/onc.2014.164 24931163 PMC4172452

[B4] Foretz M. Guigas B. Bertrand L. Pollak M. Viollet B. (2014). Metformin: from mechanisms of action to therapies. Cell Metab. 20 (6), 953–966. 10.1016/j.cmet.2014.09.018 25456737

[B5] Fu J. Lu L. Li M. Guo Y. Han M. Guo Y. (2023). A γ-Glutamyl transpeptidase (GGT)-triggered charge reversal drug-delivery system for cervical cancer treatment: *in vitro* and *in vivo* investigation. Pharmaceutics 15 (5), 1335. 10.3390/pharmaceutics15051335 37242579 PMC10221838

[B6] Hardie D. G. Ross F. A. Hawley S. A. (2012). AMPK: a nutrient and energy sensor that maintains energy homeostasis. Nat. Reviews. Mol. Cell Biology 13 (4), 251–262. 10.1038/nrm3311 22436748 PMC5726489

[B7] Herzig S. Shaw R. J. (2018). AMPK: guardian of metabolism and mitochondrial homeostasis. Nat. Reviews. Mol. Cell Biology 19 (2), 121–135. 10.1038/nrm.2017.95 28974774 PMC5780224

[B8] Hsu C. Peng D. Cai Z. Lin H. (2022). AMPK signaling and its targeting in cancer progression and treatment. Semin. Cancer. Biol. 85, 52–68. 10.1016/j.semcancer.2021.04.006 33862221 PMC9768867

[B9] Jafarzadeh E. Montazeri V. Aliebrahimi S. Sezavar A. H. Ghahremani M. H. Ostad S. N. (2022). Combined regimens of cisplatin and metformin in cancer therapy: a systematic review and meta-analysis. Life Sci. 304, 120680. 10.1016/j.lfs.2022.120680 35662589

[B10] Kang Y. J. Song W. Lee S. J. Choi S. A. Chae S. Yoon B. R. (2024). Inhibition of BCAT1-mediated cytosolic leucine metabolism regulates Th17 responses *via* the mTORC1-HIF1α pathway. Exp. and Molecular Medicine 56 (8), 1776–1790. 10.1038/s12276-024-01286-z 39085353 PMC11372109

[B11] Li A. Chen J. Li Q. Zeng G. Chen Q. Chen J. (2019). Alpha-glucosidase inhibitor 1-Deoxynojirimycin promotes beige remodeling of 3T3-L1 preadipocytes *via* activating AMPK. Biochem. Biophys. Res. Commun. 509 (4), 1001–1007. 10.1016/j.bbrc.2019.01.023 30654939

[B12] Li B. Chen G. Zhong H. Li T. Lin M. Wei H. (2024). γ-Glutamyl transpeptidase-activable nanoprobe crosses the blood-brain barrier for immuno-sonodynamic therapy of glioma. Nat. Commun. 15 (1), 10418. 10.1038/s41467-024-54382-z 39613729 PMC11607351

[B13] Liu Q. Yuan J. Jiang R. He L. Yang X. Yuan L. (2023). γ-Glutamyltransferase-Activatable fluoro-photoacoustic reporter for highly sensitive diagnosis of acute liver injury and tumor. Anal. Chem. 95 (3). 10.1021/acs.analchem.2c04894 36633322

[B14] Mitri A. Castellano I. (2023). Targeting gamma-glutamyl transpeptidase: a pleiotropic enzyme involved in glutathione metabolism and in the control of redox homeostasis. Free Radical Biology and Medicine 208, 672–683. 10.1016/j.freeradbiomed.2023.09.020 37739139

[B15] Peng B. Zhang S. Chan K. I. Zhong Z. Wang Y. (2023). Novel anti-cancer products targeting AMPK: natural herbal medicine against breast cancer. Mol. Basel, Switz. 28 (2), 740. 10.3390/molecules28020740 36677797 PMC9863744

[B16] Takemura K. Yuasa T. Inamura K. Amori G. Koga F. Board P. G. (2020). Impact of serum γ-Glutamyltransferase on overall survival in patients with metastatic renal cell carcinoma in the era of targeted therapy. Target. Oncol. 15 (3), 347–356. 10.1007/s11523-020-00719-9 32474759

[B17] Wang Y. An H. Liu T. Qin C. Sesaki H. Guo S. (2019). Metformin improves mitochondrial respiratory activity through activation of AMPK. Cell Rep. 29 (6), 1511–1523.e5. 10.1016/j.celrep.2019.09.070 31693892 PMC6866677

[B18] Wang G. Zhou Z. Zhao Z. Li Q. Wu Y. Yan S. (2020). Enzyme-triggered transcytosis of dendrimer-drug conjugate for deep penetration into pancreatic tumors. ACS Nano 14 (4), 4890–4904. 10.1021/acsnano.0c00974 32286784

[B19] Wen J. Yi Z. Chen Y. Huang J. Mao X. Zhang L. (2022). Efficacy of metformin therapy in patients with cancer: a meta-analysis of 22 randomised controlled trials. BMC Med. 20 (1), 402. 10.1186/s12916-022-02599-4 36280839 PMC9594974

[B20] Whitfield J. B. (2001). Gamma glutamyl transferase. Crit. Rev. Clin. Lab. Sci. 38 (4), 263–355. 10.1080/20014091084227 11563810

[B21] Yu O. H. Y. Suissa S. (2023). Metformin and cancer: solutions to a real-world evidence failure. Diabetes Care 46 (5), 904–912. 10.2337/dci22-0047 37185680

[B22] Yu F. Zhu Y. Liu Y. Qiu G. Shang X. Meng T. (2020). Poly-γ-glutamic acid derived nanopolyplexes for up-regulation of gamma-glutamyl transpeptidase to augment tumor active targeting and enhance synergistic antitumor therapy by regulating intracellular redox homeostasis. Biomater. Sci. 8 (21), 5955–5968. 10.1039/d0bm01254h 32966382

[B23] Zhou Z. Luo G. Li C. Zhang P. Chen W. Li X. (2023). Metformin induces M2 polarization *via* AMPK/PGC-1α/PPAR-γ pathway to improve peripheral nerve regeneration. Am. J. Transl. Res. 15 (5), 3778–3792. 37303686 PMC10250979

