# Supplementary material for: Gamma-glutamyl metformin inhibits renal cell carcinoma progression by activating AMPK signaling pathway
Source: Front Pharmacol. 2026 May 7;17:1807987. doi: 10.3389/fphar.2026.1807987 (PMC13189888; doi:10.3389/fphar.2026.1807987)
Supplement: Supplementary file 1 [file Supplementaryfile1.docx]

**Supporting Information for**

Gamma-Glutamyl Metformin Inhibits Renal Cell Carcinoma Progression by Activating AMPK Signalling Pathway

Zheng Lv^★,^^§,*^, Li-Song Zhang^★,†^, Awuti Aisha^&^, Tian-hang Wang ^†^, Hai-yan Chui ^†^, Shuai Tang^§^, Jiang-Hui Zhang^§^, Fan Chang^§^, Wen-Song Wu^#^, Lu-Yuan Li^†^, Zhi-Song Zhang*^,†^ and Fang-Min Chen*^,§,#^

^§^ Department of Urology, Central Hospital, Tianjin University /Tianjin Third Central Hospital, Tianjin 300170, China

^†^ State Key Laboratory of Medicinal Chemical Biology, College of Pharmacy and Tianjin Key Laboratory of Molecular Drug Research, Nankai University, Tianjin 300350, China

^*^ Tianjin Key Laboratory of Extracorporeal Life Support for Critical Diseases, Tianjin 300170, China

^&^ The Third Central Clinical College of Tianjin Medical University, Tianjin 300170, China

^#^ Department of Urology, affiliated hospital of Jianghan University, Wuhan, 430000, China

**Author Contributions**

^★^These authors contributed equally.

**Corresponding Authors:**

*E-mail: zzs@nankai.edu.cn

*E-mail: cfm0410@163.com

**S1. Experiment Materials and Instruments**

Six-well and 96-well cell culture plates were obtained from Corning Costar Co. (Cambridge, MA, USA). 1640 medium, minimal essential medium, high-glucose DMEM, fetal bovine serum (FBS) and 1% penicillin/streptomycin (PS) were bought from Gibco (Grand Island, NY, USA). Cell counting kit-8 (CCK8) and Hematoxylin-eosin (HE) staining kit were purchased from Beyotime (Shanghai, China). The purified deionized water was prepared by the Milli-Q plus system (Millipore Co., Billerica, MA, USA). Human pCMV3-GGT plasmid were purchased from SinoBiological technology (Beijing, China). 293T cells, human renal epithelial tubular cell line HK-2, the human renal cancer cell line Caki-1, 769P, 786O, ACHN and mouse renal cancer cell line Renca were purchased from American Type Culture Collection (ATCC) and maintained in our lab. All Balb/C mouse (purchased from Vital River Laboratory Animal Technology Co., Ltd. Beijing, China) were used at 6 to 8 weeks old and strictly comply with animal ethics requirements. All reagents and solvents were purchased from Shanghai Bide Pharmatech Ltd. Co., unless otherwise specified, and used without further purification. Flash column chromatography employed silica gel (100−200 mesh). NMR spectra were recorded in DMSO-d6 or D2O, unless otherwise stated, on Bruker 400 (1H at 400 MHz, 13C at 100 MHz) NMR spectrometers, and chemical shifts are reported in units of δ relative to internal TMS. High-resolution mass spectra were performed in The State Key Laboratory of Medicinal Chemical Biology, College of Pharmacy, Nankai University, Nankai University. Mass spectral data are reported in the form of m/z (intensity relative to base = 100). Purities of the tested compounds were determined by HPLC analysis using Agela Technologies HPLC using Agela Technologies MP C18 columns (5 μm, 4.6 mm × 50 mm column and Methanol/water) and were > 95%. Animals were purchased from Beijing Vital River Laboratory Animal Technology Co., Ltd.

**S2. The details of syntheses of γE-Met**

Dissolve Met (1.29g, 10mmol) and (S)-5- (term-butoxy)-4-(term-butoxycarbonyl) amino) -5-oxopentanoid acid (3.03g, 10mmol) in anhydrous pyridine solution, and phosphorus oxychloride (1.53g, 10mmol). After stirring at room temperature for 1 hour, remove pyridine from the mixture by rotary evaporation and extract with ethyl acetate. The crude product was purified by silica gel column chromatography, and methanol/dichloromethane (1:50, v/v) was used as the eluent to obtain a white solid Boc-γE-Met (1.65g, yield: 39.86 %). Dissolve Boc-γE-Met (4.14g, 10mmol) in 95% trifluoroacetic acid solution and react at room temperature for 30 minutes. Remove trifluoroacetic acid and dichloromethane from the mixture by rotary evaporation, and purify the crude product by high-performance liquid chromatography to obtain a white solid γE-Met (1.36g, 52.71%).

**S3. Supplementary Tables.**

**Supplementary table 1** The relationship between GGT expression and clinicopathological features in patients with ccRCC (n=125)

| Variable | No. of patients (%) | | | *χ^2^* | *P* value |
| --- | --- | --- | --- | --- | --- |
|  | Patients | GGT1 positive（n=60） | GGT1 negative (n=65) |  |  |
| Age (years) |  |  |  |  |  |
| ≤60 | 34 | 22（36.67） | 12（18.46） | 5.222 | 0.022 |
| > 60 | 91 | 38（63.33） | 53（81.54） |  |  |
| Gender |  |  |  |  |  |
| Male | 98 | 45（75.00） | 53（81.54） | 0.788 | 0.375 |
| Female | 37 | 15（25.00） | 12（18.46） |  |  |
| T stage |  |  |  |  |  |
| T1+T2 | 99 | 41（68.33） | 58（89.23） | 8.271 | 0.004 |
| T3+T4 | 26 | 19（31.67） | 7（10.77） |  |  |
| N stage |  |  |  |  |  |
| N0 | 116 | 55（91.67） | 61（93.85） | 0.222 | 0.638 |
| N1 | 9 | 5（8.33） | 4（6.15） |  |  |
| M stage |  |  |  |  |  |
| M0 | 109 | 45（75.00） | 64（98.46） | 15.387 | 0.000 |
| M1 | 16 | 15（15.00） | 1（1.54） |  |  |
| AJCC Stage |  |  |  |  |  |
| Stage Ⅰ+Ⅱ | 87 | 31（51.67） | 56（86.15） | 17.538 | 0.000 |
| Stage Ⅲ+Ⅳ | 38 | 29（48.33） | 9（13.85） |  |  |
| Fuhrman grade |  |  |  |  |  |
| Grade 1+2 | 83 | 30（50.00） | 53（81.54） | 13.910 | 0.000 |
| Grade 3+4 | 42 | 30（50.00） | 12（18.46） |  |  |

**Supplementary Table 2.**  Antibodies

| **Antibodies** |  |  |  |  |
| --- | --- | --- | --- | --- |
| Name | Manufacturer | Number | Type | Usage |
| GGT1 | SANTA CRUZ | sc-100746 | Monoclonal | WB,IHC,IP,IF |
| AMPKα1/2 | Abcam | ab107442 | Monoclonal | IP,WB |
| p-AMPKα1/2 | Abcam | ab183448 | Monoclonal | WB |
| mTOR | SANTA CRUZ | sc-517464 | Monoclonal | WB,IP,IF |
| p-mTOR | SANTA CRUZ | sc-293133 | Monoclonal | WB,IP,IF,IHC |
| β-actin | Abcam | ab178787 | Monoclonal | WB |
| HIF-1α | Abcam | ab179483 | Monoclonal | WB,IF,CHIP |
| VEGFA | Abcam | ab46154 | Polyclonal | WB |
| MDR1 | SANTA CRUZ | sc-13131 | Monoclonal | WB, IP,IF,IHC |
| GLUT1 | SANTA CRUZ | sc-377228 | Monoclonal | WB,IP,IF,IHC |
|  |  | | | |

**Supplementary table 3** Univariate and multivariable Cox regression models analyzing clinical variables affecting OS and PFS.

|  | **OS** | | | |  | **PFS** | | | |
| --- | --- | --- | --- | --- | --- | --- | --- | --- | --- |
| Variable | Univariate | | Multivariate | |  | Univariate | | Multivariate | |
|  | *HR (95% CI)* | *P* | *HR (95% CI)* | *P* |  | *HR (95% CI)* | *P* | *HR (95% CI)* | *P* |
| Age(years) 1:＜60；0：≥60 | 0.417(0.201～0.868) | 0.023 | 0.625(0.267～1.464) | 0.279 |  | 0.804(0.370～1.747) | 0.581 | 1.820(0.723～4.580) | 0.204 |
| Gender 1:male；0：female | 0.838(0.358～1.961) | 0.683 | 0.837(0.303～2.331) | 0.731 |  | 0.932(0.402～2.164) | 0.870 | 1.999(0.678～5.899) | 0.210 |
| BMI 1:＜23.9；0:≥23.9 | 1.613(0.762～3.416) | 0.212 | 1.317(0.559～3.104) | 0.529 |  | 2.103(1.027～4.306) | 0.042 | 2.365(1.008～5.548) | 0.048 |
| T stage 1: ≥T_3_；0:＜T_3_ | 3.584(1.691～7.599) | 0.001 | 0.662(0.176～2.490) | 0.542 |  | 4.145(2.027～8.475) | 0.000 | 0.377(0.084～1.704) | 0.205 |
| N stage 1:N_1_；0: N_0_ | 4.218(1.592～11.175) | 0.004 | 2.431(0.620～9.526) | 0.202 |  | 4.983(2.021～12.287) | 0.000 | 4.577(1.236～16.948) | 0.023 |
| M stage 1:M_1_；0: M_0_ | 9.237(4.141～20.602) | 0.000 | 1.827(0.443～7.536) | 0.404 |  | 5.608(2.438～12.899) | 0.000 | 1.232(0.316～4.800) | 0.764 |
| AJCC 1: ≥Ⅲ；0:＜Ⅲ | 8.480(3.730～19.277) | 0.000 | 3.875(0.617～24.345) | 0.149 |  | 6.376(3.046～13.345) | 0.000 | 4.730(0.796～28.098) | 0.087 |
| Fuhrman grade 1: ≤2；0:＞2 | 6.887(3.041～15.593) | 0.000 | 2.774(1.110～6.934) | 0.029 |  | 4.911(2.331～10.347) | 0.000 | 1.985(0.833～4.725) | 0.122 |
| GGT 1:positive；0: negative | 9.047(3.136～26.101) | 0.000 | 4.194(1.293～13.601) | 0.017 |  | 9.274(3.502～24.554) | 0.000 | 6.867(2.369～19.905) | 0.000 |

**S4. Supplementary Figures.**


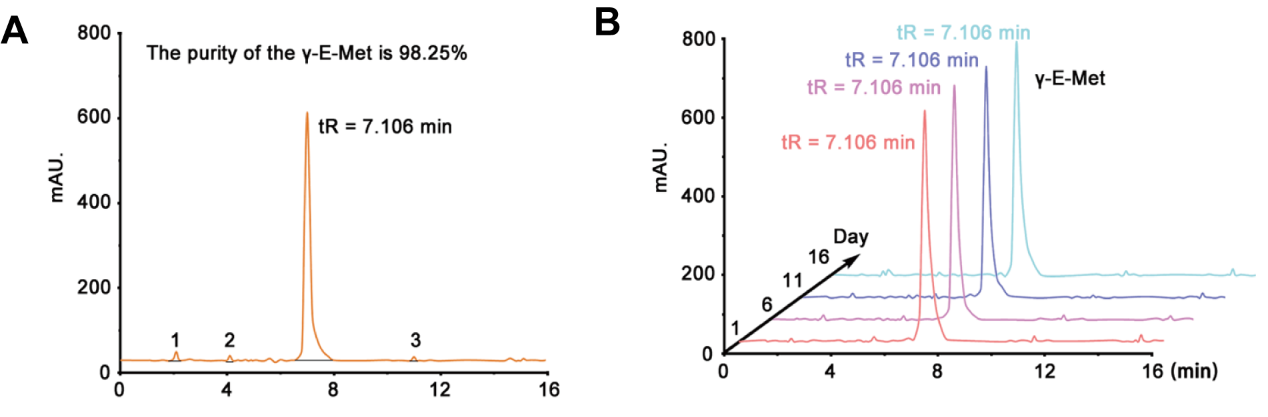


**Figure S1.** (A) Purity Analysis of γ-E-metformin by High Performance Liquid Chromatography (HPLC), tR = 7.106 min. (B)Stability testing of γ-E-metformin at different time points (day 1, day 6, day 11, and day) using high-performance liquid chromatography (HPLC), tR = 7.106 min. The sample maintained stable chemical structure for 16 days

**
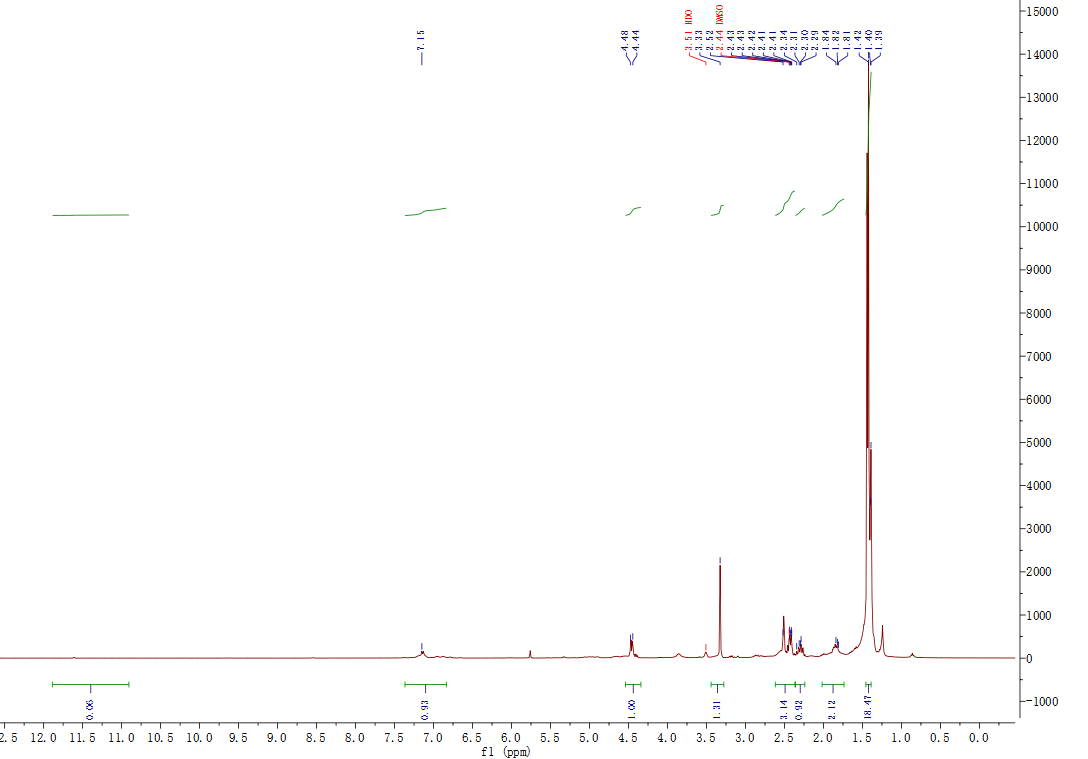
**

**Figure S2.** ^1^H NMR spectrum (400 MHz) of Boc-γE-Met in DMSO-d6 at 25 ºC.

**
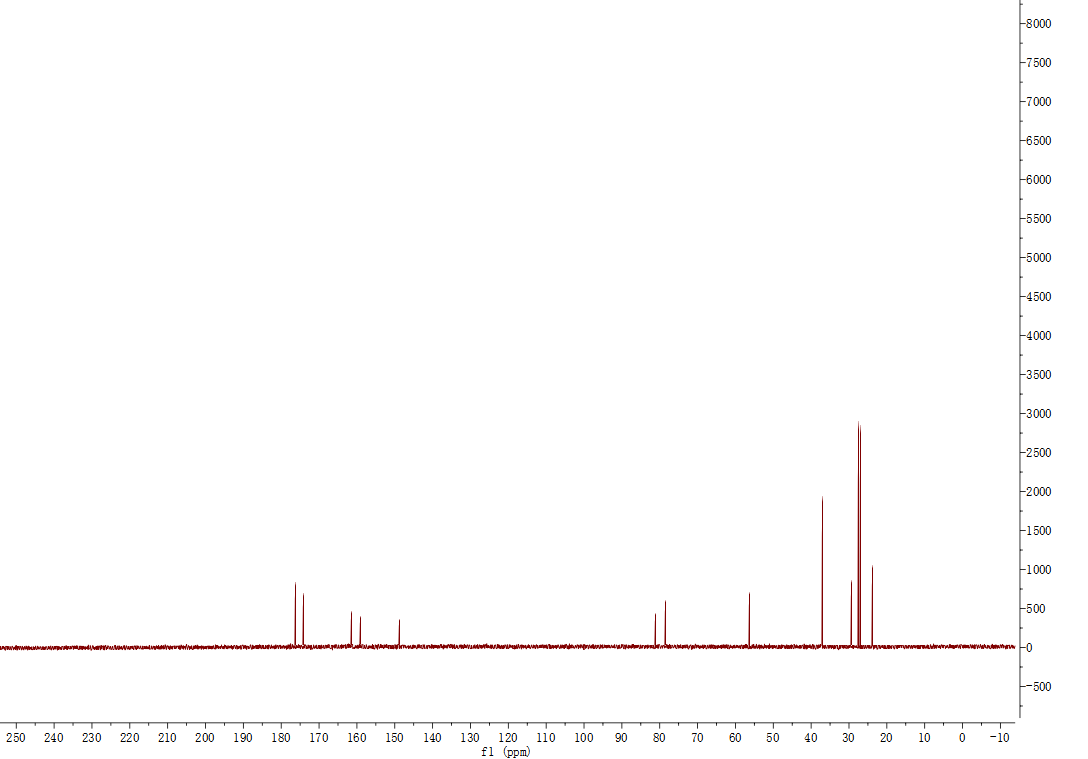
**

**Figure S3.** ^13^C NMR spectrum (400 MHz) of Boc-γE-Met in D_2_O at 25 ºC.

**
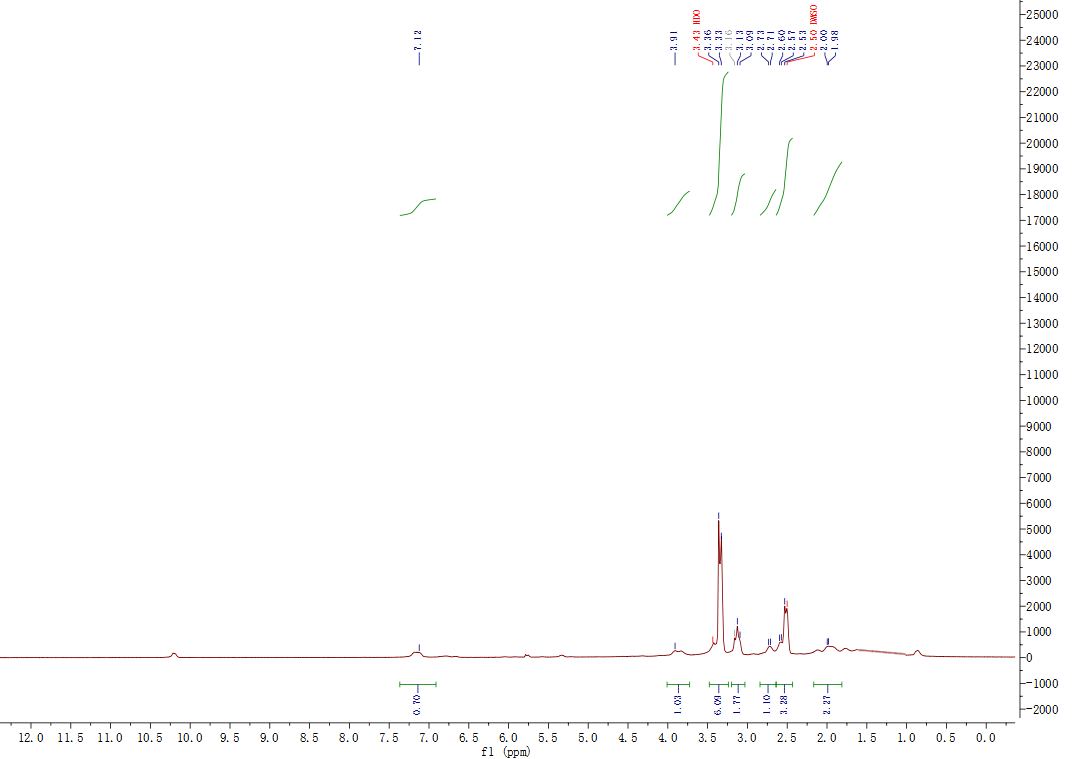
**

**Figure S4.** ^1^H NMR spectrum (400 MHz) of γE-Met in DMSO-d6 at 25 ºC.

**
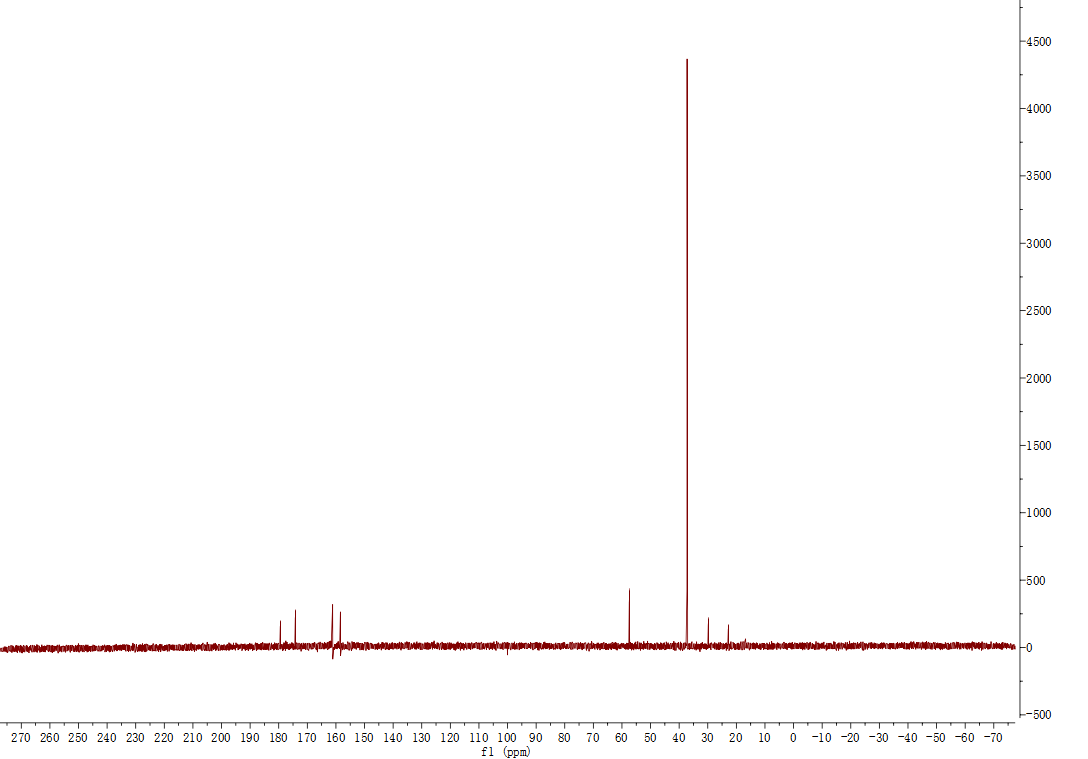
**

**Figure S5.** ^1^H NMR spectrum (400 MHz) of γE-Met in D2O at 25 ºC.

**
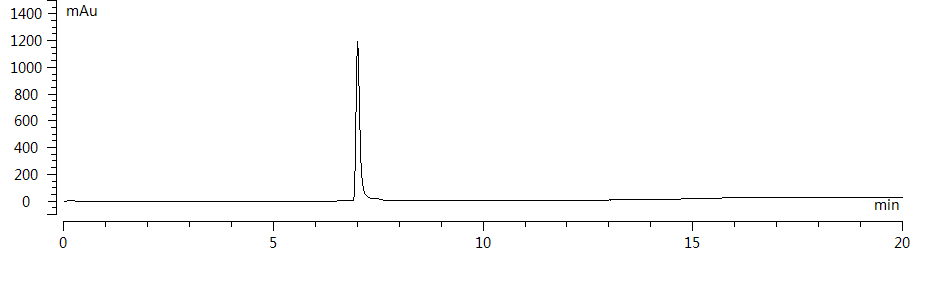
**

**Figure S6.** Liquid phase chromatogram of γE-Met in MCN/H2O.

**
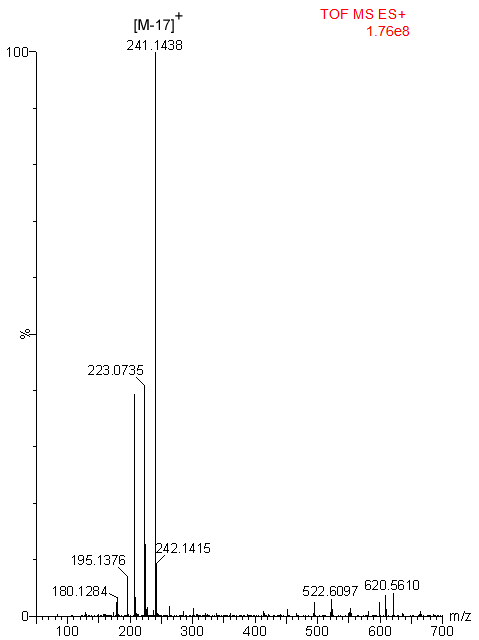
**

**Figure S7.** HR-MS spectrum of γE-Met. M-17=241.1438.


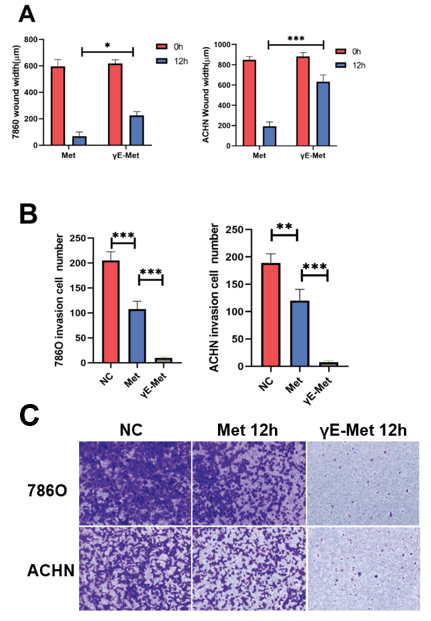


**Figure S8.** The anti-cancer activity of Met and γE-Met on renal cancer cells (786O, ACHN) in vivo. (A)The histogram of the migration ability of renal cancer cells after incubated with Met and γE-Met for 12h. *p< 0.05, ***p < 0.001. (B) The histogram of the invasion ability of renal cancer cells after incubated with Met and γE-Met for 12h. ***p < 0.001.


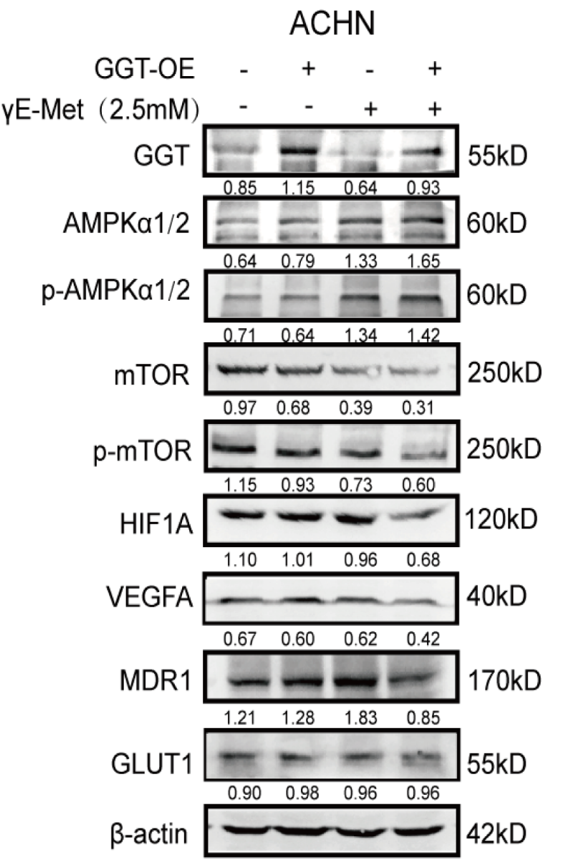

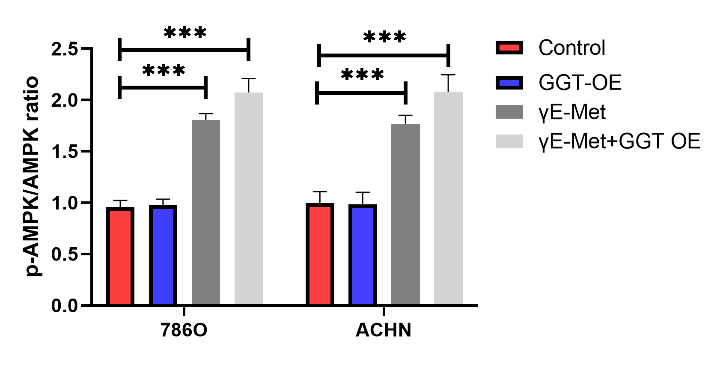


**Figure S9.** the levels of AMPK/mTOR signaling pathway related proteins, including AMPK, p-AMPK (Thr172), mTOR, p-mTOR (Ser2448) , HIF1A, VEGFA, MDR1 and GLUT1 were determined by WB analysis in ACHN cells treated with γE-Met and/or GGT overexpression (left). The histogram of p-AMPK/AMPK ratio in each group (left). ***P<0.001.


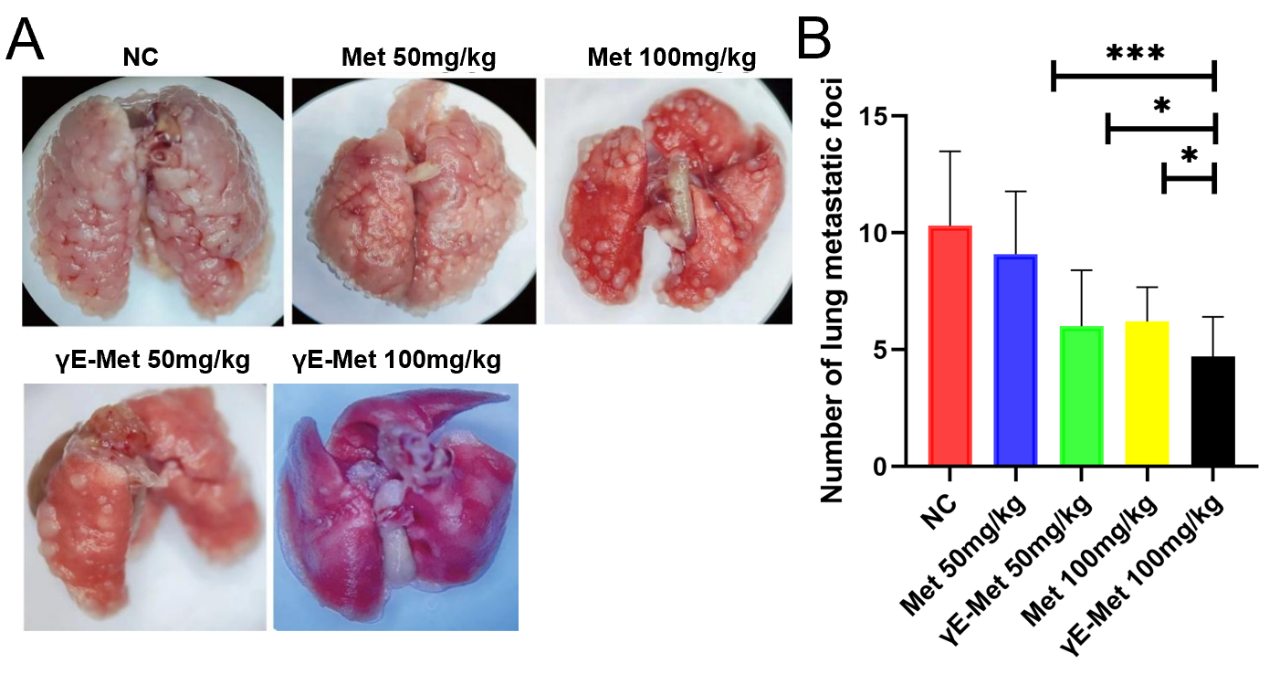


**Figure S10.** The gross appearance of lung metastasis foci in NC group, Met 50/100mg group, γE-Met 50/100mg group. (A) the light image of gross appearance of lung metastasis foci in NC group, Met 50/100mg group, γE-Met 50/100mg group.


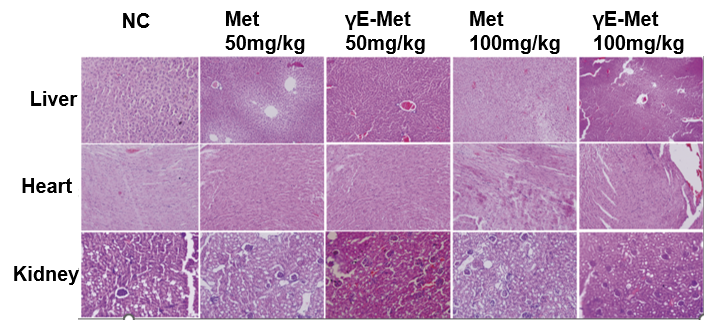


**Figure S11.** Pathological changes in the liver, heart and kidney were detected by HE staining.


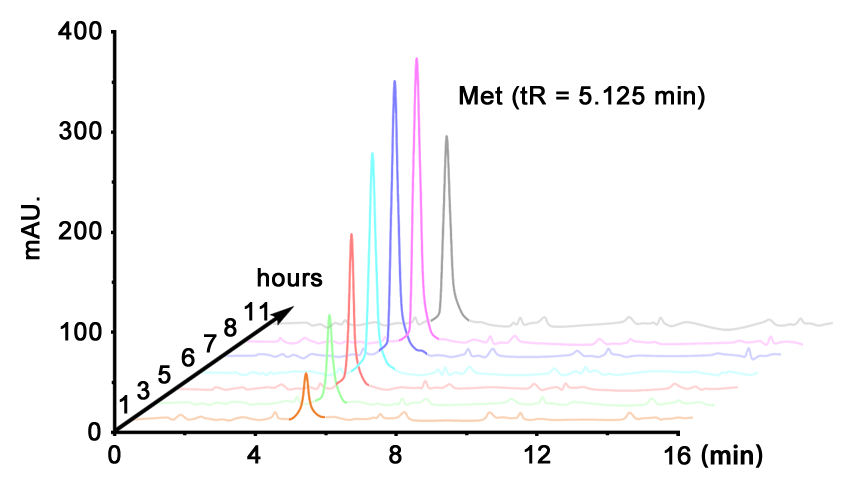


**Figure S12.** Detecting the efficiency of γE-metformin releasing metformin at different time points (1, 3, 5, 7, 8, and 11 hours) under the action of γ-glutamyl transferase (GGT) in tumor tissue using high-performance liquid chromatography (HPLC), tR = 5.125 min.


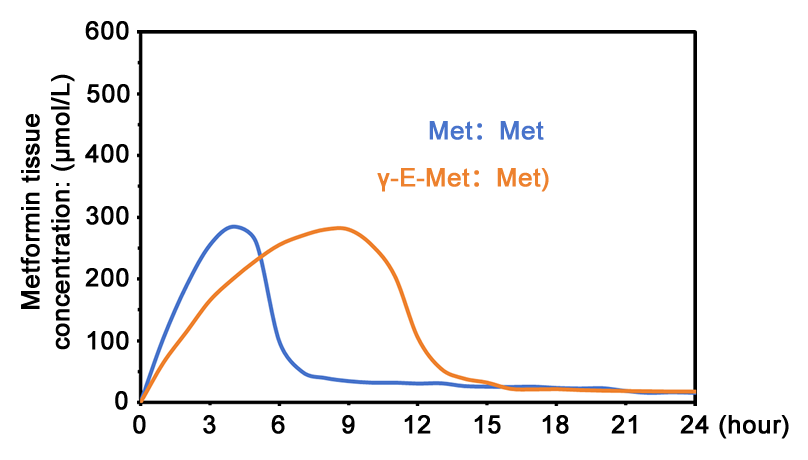


**Figure S13.** The mean concentration-time curves of metformin in tumor tissue and metformin released from γE-metformin in tumor tissue, respectively (0-24 hours). Compared with the use of metformin, metformin released by γE-metformin at the same concentration maintained a longer treatment time in tumor tissue.


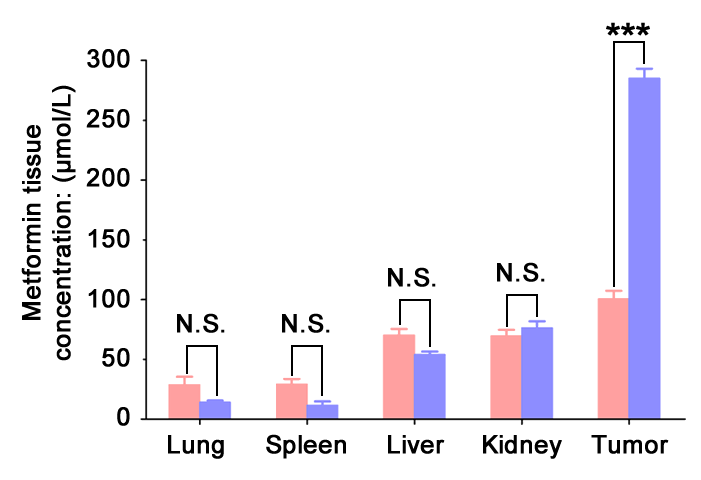


**Figure S14.** Tissue Met concentrations (tumor, kidney, liver, spleen, and lung) 24 hr post‐administration of γE-Met/Met 100 mg group.


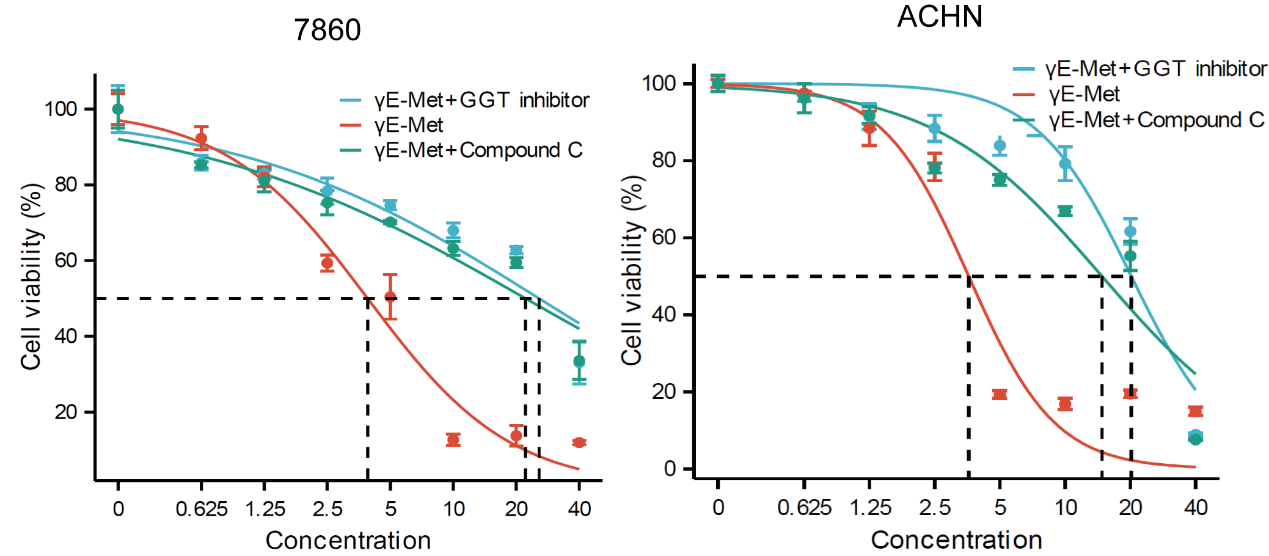


**Figure S15.** Renal cancer cells were incubated with γE-Met, γE-Met+GGT inhibitor and γE-Met+Compound C for 24h and then subjected to CCK8 assays.


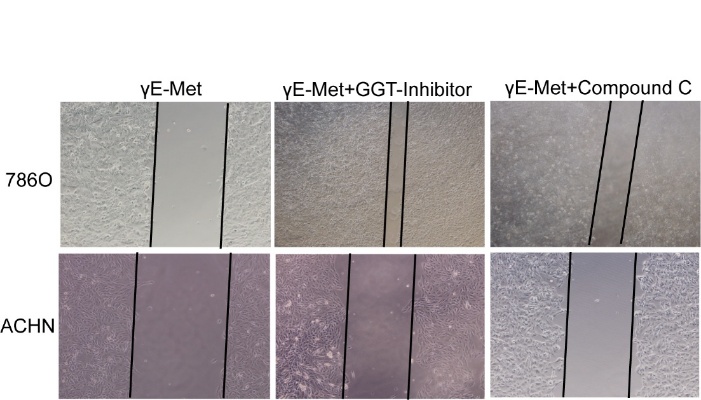

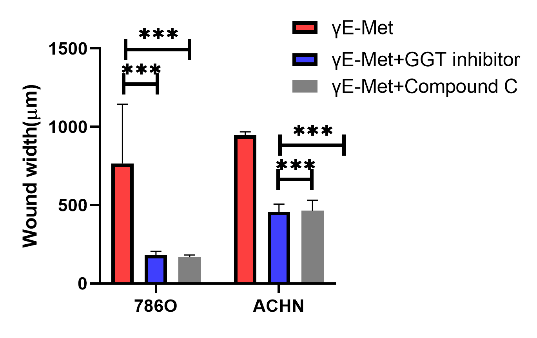


**Figure S16.** The migration ability of renal cancer cells after incubated with γE-Met, γE-Met+GGT inhibitor and γE-Met+Compound C for 12h by Scratch assays. Light image (left), the histogram of the migration ability (right).


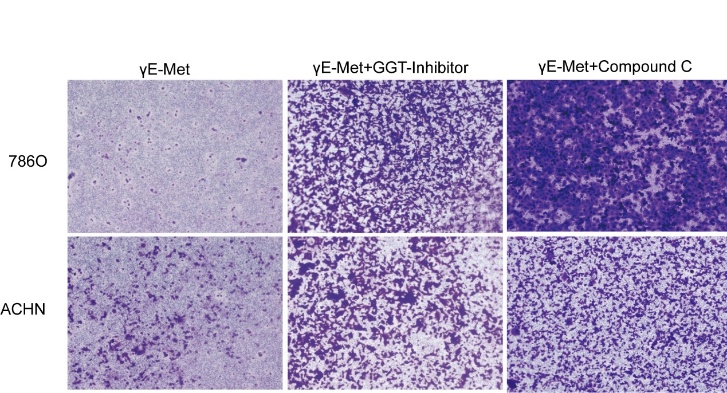

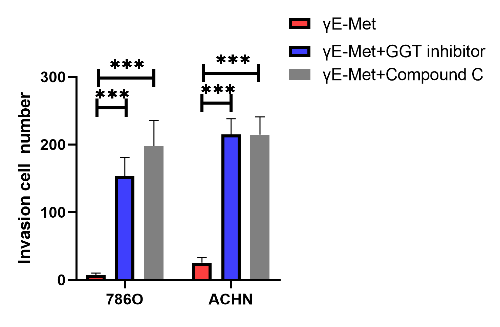


**Figure S17.** The invasion ability of renal cancer cells after incubated with γE-Met, γE-γE-Met+GGT inhibitor and γE-Met+Compound C for 12h by Transwell assays. Light image (left), the histogram of the invasion ability (right).


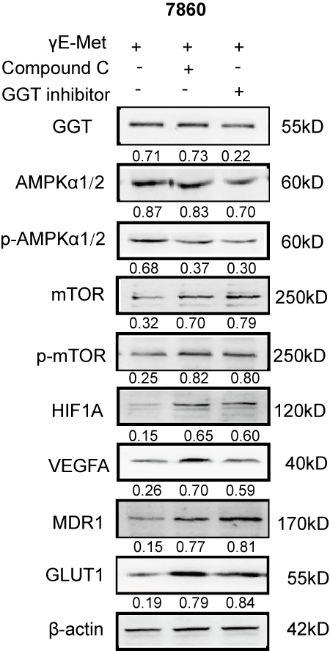

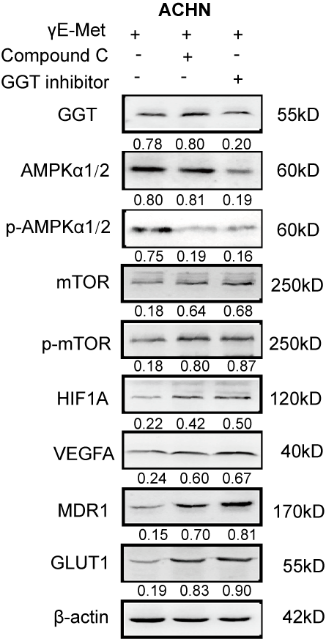


**Figure S18.** The levels of AMPK signaling pathway related proteins were determined by WB analysis in 786O or ACHN treated with γE-Met, Met+GGT inhibitor and Met+Compound C.


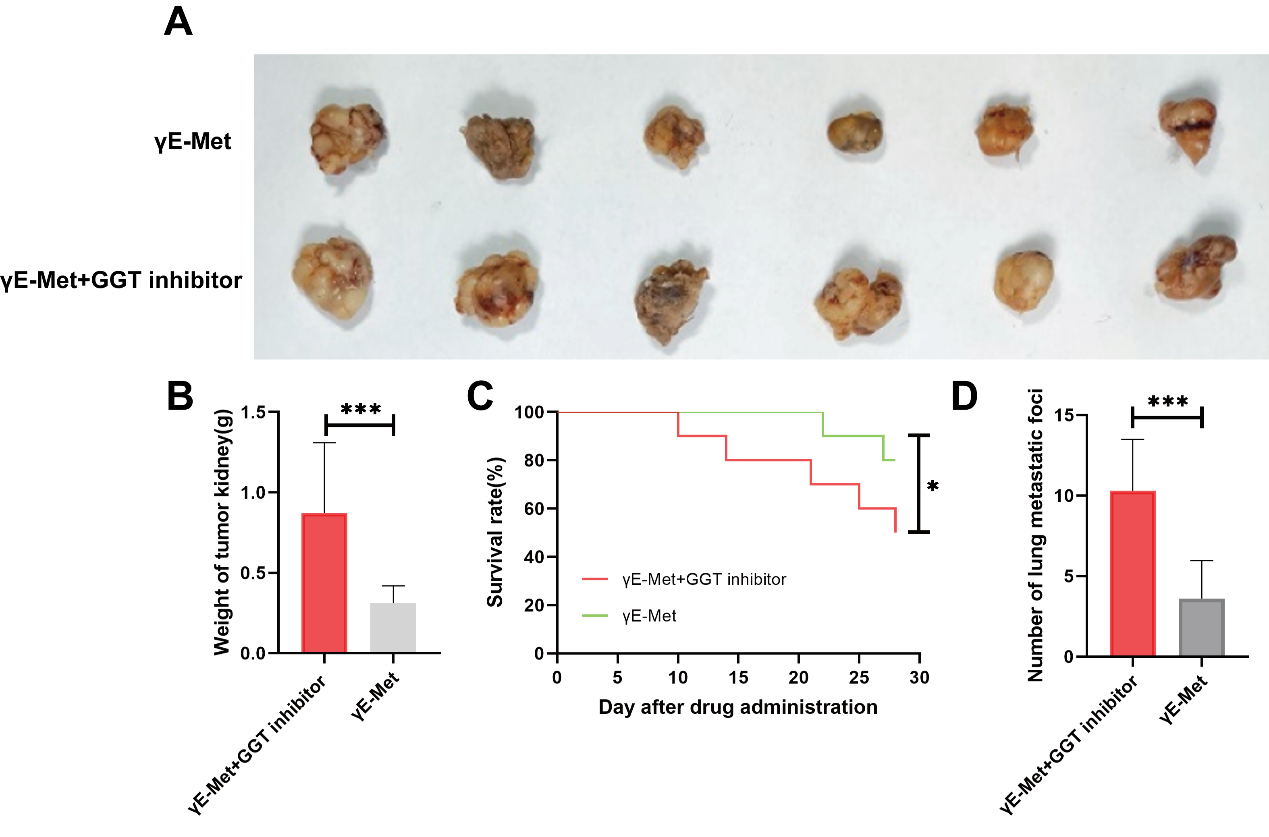


**Figure S19.** (A) Gross appearance of kidney tumors in groups of γE-Met and γE-Met+GGT inhibitor. (B) The weight of tumor kidney in groups of γE-Met and γE-Met+GGT inhibitor were compared. ***P<0.001. (C) The survival rates of mice in each group were compared by Kaplan-Meier analysis. *p< 0.05. (D) The number of lung metastatic foci were compared. ***P<0.001.
